# Lotus Leaf Aqueous Extract Reduces Visceral Fat Mass and Ameliorates Insulin Resistance in HFD-Induced Obese Rats by Regulating PPARγ2 Expression

**DOI:** 10.3389/fphar.2017.00409

**Published:** 2017-06-23

**Authors:** Kemin Yan, Huijuan Zhu, Jian Xu, Hui Pan, Naishi Li, Linjie Wang, Hongbo Yang, Meijuan Liu, FengYing Gong

**Affiliations:** ^1^Key Laboratory of Endocrinology of National Health and Family Planning Commission, Department of Endocrinology, Peking Union Medical College Hospital, Chinese Academy of Medical Science and Peking Union Medical CollegeBeijing, China; ^2^Department of Endocrinology, Beijing Tian Tan Hospital, Capital Medical UniversityBeijing, China

**Keywords:** lotus leaf aqueous extract (LLAE), PPARγ2, visceral adipose tissue, insulin resistance, obese rats

## Abstract

**Objectives:** Lotus leaf is a kind of traditional Chinese medicine. We aimed to explore the effects of lotus leaf aqueous extract (LLAE) on peroxisome proliferative activated receptor γ2 (PPARγ2) expression in preadipocytes and adipocytes and further investigate its effects on high fat diet (HFD)-induced obese rats.

**Methods:** pGL3-Enhancer-PPARγ2 (625 bp)-Luc plasmid, a luciferase reporter gene expression plasmid containing PPARγ2 promoter, was stably transfected into 3T3-L1 preadipocytes. PPARγ2 promoter activities were determined by assaying the luciferase activities. Then PPARγ2 promoter activities in preadipocytes and PPARγ2 mRNA levels in human subcutaneous adipocytes were measured after the administration with LLAE. Additionally, the effects of LLAE on body weight, fat mass, glucose and lipid metabolism and the expression of PPARγ2, insulin receptor substrate 1 and glucose transporter 4 (GLUT4) in visceral adipose tissue (VAT) were measured in HFD-induced obese rats treated with low or high dose [0.5 or 3.0 g crude drug/(kg.d)] LLAE for 6 weeks.

**Results:** Ten μg/ml LLAE significantly increased the luciferase activities in 3T3-L1 cells and the stimulatory action reached 2.51 folds of controls when LLAE was 1000 μg/ml (*P* < 0.01). After treating 3T3-L1 cells with 100 μg/ml LLAE, the stimulatory role peaked at 32 h where it was 2.58 folds of controls (*P* < 0.01). Besides, 100 μg/ml LLAE significantly increased PPARγ2 mRNA levels in human adipocytes to 1.91 folds of controls (*P* < 0.01). In HFD-induced obese rats, administration with both low and high dose LLAE notably reduced visceral fat mass by 45.5 and 58.4%, respectively, and significantly decreased fasting serum insulin levels (*P* < 0.05). The high dose LLAE also significantly decreased homeostasis model assessment of insulin resistance in obese rats (*P* < 0.05). Furthermore, the mRNA levels of PPARγ2 and GLUT4 in VAT of obese rats were significantly increased when compared with control rats, and were notably suppressed by LLAE intervention for 6 weeks (*P* < 0.05).

**Conclusion:** LLAE significantly reduces visceral fat mass and ameliorates insulin resistance in HFD-induced obese rats. These beneficial effects of LLAE may associate with its role in stimulating PPARγ2 expression in preadipocytes and subcutaneous adipocytes and suppressing PPARγ2 and GLUT4 expression in VAT.

## Introduction

Obesity is a chronic metabolic disease caused by many factors, which derives from an imbalance between energy intake and expenditure and finally leads to excessive and ectopic fat accumulation in our body. It is becoming a major risk factor for many diseases such as type 2 diabetes, cardiovascular diseases, and several kinds of tumors (Djalalinia et al., 2015). The study of obesity has attracted the attention of scholars all over the world. However, drugs for obesity treatment are limited and have many adverse reactions (Jones and Bloom, 2015).

Some TCMs have been reported to be useful and safe in the treatment of obesity by acting on multiple targets (Yin et al., 2008; Hasani-Ranjbar, 2009; Gu and Huang, 2013). Lotus leaf, also known as Nelumbo nucifera leaf and is rich in a number of alkaloids such as nuciferine, pronuciferine, roemerine and so on, is such kind of TCMs with many beneficial roles in organism (Mukherjee et al., 2009; Sharma et al., 2017), including anti-obesity (Ono et al., 2006; Ahn et al., 2013), antioxidant (Lin et al., 2009; Zhu et al., 2015), hypoglycemic and hypolipidemic effects (Kim et al., 2013; Zeng et al., 2016), and lipolytic activity (Siegner et al., 2010). The anti-obesity effect of lotus leaf was reported to be associated with inhibiting the activities of pancreatic alpha-amylase and lipase *in vitro* (Ono et al., 2006; Ahn et al., 2013), accelerating lipid metabolism and up-regulating energy expenditure in HFD-induced obese mice and rats (Ono et al., 2006) and suppressing adipocyte differentiation in 3T3-L1 preadipocytes (Ahn et al., 2013). However, the detailed mechanism by which lotus leaf loses weight is largely still unknown.

Peroxisome proliferative activated receptors, which belong to the thyroid/retinoid nuclear receptor family, have been reported to control the expression of genes involved in adipogenesis in preadipocytes differentiation process (Mangelsdorf et al., 1995; Wang et al., 2014). PPARγ, especially the PPARγ2 isoform, is predominately expressed in adipose tissue and plays a significant role in adipogenesis, insulin sensitization and homeostasis of lipid and glucose (Willson et al., 2001; Feng et al., 2016). Adipose-specific activation of PPARγ is sufficient to reverse whole body insulin resistance to a similar degree as systemic TZD treatment (Sugii et al., 2009). TZD, a PPARγ agonist, has been reported to decrease the blood glucose levels and ameliorate insulin resistance in diabetic animal model and patients and has been widely used in clinical practice (Saraf et al., 2012; Janani and Ranjitha Kumari, 2015). Interestingly, Guo et al. (2013) reported that nuciferine, a chemical composition of lotus leaf, could also prevent hepatic steatosis and injury induced by a HFD in hamsters through suppressing the expression of PPARγ in liver. However, whether lotus leaf could affect the expression of PPARγ in adipocytes and adipose tissue has not been elucidated yet.

Therefore, in the present study, we aimed to explore the effects of LLAE on PPARγ2 expression in 3T3-L1 preadipocytes and human subcutaneous adipocytes differentiated from preadipocytes, and further investigate its effects on body weight, fat mass, insulin resistance and PPARγ2 expression in VAT of HFD-induced obese rats.

## Materials and Methods

### Preparation of LLAE

Lotus leaf (100 g, purchased from Beijing Tongrentang pharmaceuticals company, Beijing, China) was soaked in 300 ml cold water for 2 h, then was boiled for 30 min and filtered with eight layers of gauzes. Then 200 ml water was added to the residue and the mixture was boiled and filtered again as above. The two filtrates were mixed thoroughly and centrifuged at 4000 rpm for 20 min. The supernatant was taken and freeze-dried. The powder obtained was LLAE. 4 g LLAE was dissolved in 4 ml deionized water to make 1000 mg/ml LLAE stock solution.

### Construction of pGL3-Enhancer-PPARγ2 (625 bp)-Luc Plasmid

pGL3-Enhancer-PPARγ2 (625 bp)-Luc plasmid was constructed as described previously (Zhu et al., 2013). In brief, pGL3-PPARγ2 (625 bp)-Luc, which contained the human PPARγ2 gene 5′-promoter fragment spanning -615 to +10 bp (+1 indicates the transcription start site) and was constructed previously in our laboratory, was digested by the restriction enzymes KpnI and BglII (Takara, Japan) to get the 625 bp PPARγ2 gene promoter inserting fragment. The fragment was then inserted into pGL3-Enhancer-Luc vector to yield pGL3-Enhancer-PPARγ2 (625 bp)-Luc plasmid. An electrophoresis was conducted to verify the constructed plasmid, and the plasmid was further sequenced by Bioasia Biotechnology Company (Shanghai, China).

### Stable Transfection in 3T3-L1 Preadipocytes

Stably transfected 3T3-L1 preadipocytes were created as described previously (Gong et al., 2006). In brief, 3T3-L1 preadipocytes (obtained as a generous gift from Department of genetics, Institute of life sciences, Peking University, Beijing, China) were cultured with Dulbecco’s modified Eagle’s-F12 (DMEM/F12, Hyclone, Logan, UT, United States) medium supplemented with 10% FBS, then were plated in 12-well plates at a density of 4.5 × 10^5^ cells/well. The cells were co-transfected with 1.2 μg pGL3-Enhancer-PPARγ2 (625 bp)-Luc and 0.4 μg pcDNA3.1(+), which carried the neomycin resistance gene, and 4 μl lipofectamine2000 (Invitrogen, Carlsbad, CA, United States) at a ratio of weight/volume 1/2.5 in 0.4 ml OPTI-MEM medium for 5 h. Then cells were incubated with medium containing 300 μg/ml Geneticin G418 (Invitrogen, Carlsbad, CA, United States) to screen the monoclonal cells. The luciferase activity was measured using commercially available Luciferase Assay System kit (Promega, Madison, WI, United States) in microplate luminescence analyzer (Beijing Hamamatsu Photon Techniques, inc., Beijing, China) as described previously (Zhu et al., 2014). The relative luciferase activity of stably transfected 3T3-L1 preadipocytes, which were transfected with pGL3-Enhancer-PPARγ2 (625 bp)-Luc plasmid, was obtained in comparison with that of cells transfected with blank plasmid. The three appropriate colonies which expressed luciferase in a middle degree were chosen for the following studies.

### LLAE Stimulation Experiments

Stably transfected 3T3-L1 preadipocytes were seeded in 12-well plates at a density of 2.5 × 10^5^ cells/well. Then cells were treated with different concentrations (1 ∼ 1000 μg/ml) of LLAE for 18 h or were treated with 100 μg/ml LLAE for different time (2 ∼ 36 h). Then cells were lysed and the luciferase activities were measured. The relative luciferase activity of LLAE treated cells was obtained in comparison with that of controls. The experiments were performed in three different colonies. Samples in each experiment were in triplicate.

### Human Primary Preadipocytes Differentiation and RT-PCR Analysis

Human primary preadipocytes [obtained from the abdominal subcutaneous adipose tissues (SATs) of three patients with benign diseases (appendicitis, cholelithiasis, and colon benign neoplasm) and preserved in our laboratory] were differentiated as previously described (Zhu et al., 2006). Briefly, cells seeded in six-well plates at a density of 5 × 10^4^ cells/well were grown to 90% confluence, and then were induced to differentiate in serum-free DMEM/F12 medium supplemented with 0.5 μM insulin (Sigma–Aldrich, United States), 0.25 μM dexamethasone (Sigma–Aldrich, United States), 0.2 nM thyroxine (Sigma–Aldrich, United States) and 0.5 nM 3-isobutyl-1-methylxanthine (Sigma–Aldrich, United States) for 6 days. The fully differentiated cells were then administrated with 100 μg/ml LLAE for 36 h. The total RNA was extracted using an EZNA total RNA kit (Omega Bio-Tek, Doraville, GA, United States), and 0.5 μg RNA was reverse transcribed with a SuperScript First-Strand Synthesis System Kit (Invitrogen, Carlsbad, CA, United States). 2 μl RT products (cDNA) were amplified with PPARγ2 primers or β-actin primers as shown in Supplementary Table S1. Amplification was carried out as follows: 10 min at 95°C, 35×(1 min at 94°C, 1 min at 59°C, and 1 min at 70°C), and 10 min at 72°C. The predicted products of PPARγ2 and β-actin were 223 and 540 bp, respectively. 10 μl RT-PCR products were electrophoresed in 2% agarose gel in Tris-acetate-EDTA buffer. The gel was then stained with ethidium bromide and photographed using Alphalmager M2200 (AlphaInnotech, San Leandro, CA, United States). The density ratio of PPARγ2 to β-actin PCR products was calculated to evaluate the PPARγ2 expression changes. The experiments were repeated for three times. Samples in each experiment were in triplicate. The study was approved by the ethics committee of Peking Union Medical College Hospital (No. JS-1093). All patients signed informed consent before being taken the adipose tissue during the surgery.

### Animals

Four-week-old male Sprague Dawley (SD) rats (weight 45 ± 1.5 g) were purchased from Beijing Vital River Laboratory Animal Technology Company (Beijing, China). All rats were housed in standard cages as in previous animal experiments (Zhu et al., 2016). Rats were randomly divided into SF group (*n* = 8) and HFD group (*n* = 22) which was further divided into simple HFD group (*n* = 7), low dose LLAE intervention group (LLLAE, *n* = 8) and high dose LLAE intervention group (HLLAE, *n* = 7). The composition of the experimental diet (Beijing HFK Bioscience, Co. Ltd, Beijing, China) was shown in Supplementary Table S2. Rats in LLLAE and HLLAE groups were administrated with 0.5 g crude drug/(kg.d) and 3.0 g crude drug/(kg.d) LLAE, respectively, by gavages. Rats in SF and HFD groups were given gavages with the same volume of saline. All rats were weighted every 5 ∼ 6 days and the dose of LLAE was changed according to body weight change. Food intake was calculated by subtracting the amount of residue food from the amount of supply food. After 6 weeks of LLAE intervention, rats were anesthetized with 10% urethane (1.0 g/kg) after a 12-h fasting. Blood samples were drawn from abdominal aorta and centrifuged immediately at 3000 rpm for 10 min at 4°C. VAT including epididymal white adipose tissue (eWAT) and perirenal white adipose tissue (pWAT) was dissected and weighted, then was immediately frozen in liquid nitrogen and stored at -80°C. Serum fasting blood glucose (FBG), total cholesterol (TC), triglycerides (TG), low density lipoprotein-cholesterol (LDL-c), and high density lipoprotein-cholesterol (HDL-c) were measured by routine automated laboratory methods. Serum insulin levels were measured by insulin radioimmunoassay kit (Linco, United States). HOMA-IR was calculated according to the following formula: fasting serum insulin (pmol/L) × FBG (mmol/L)/135 (Nordstrand et al., 2013). RT-qPCR analysis was performed in VAT using SYBR Premix Ex Taq (Takara, Japan) as previously described (Zhu et al., 2016). In brief, total RNA was extracted from adipose tissue using RNeasy Lipid Tissue Mini Kit (Qiagen, Germany) according to the supplier’s instructions. Then 1 μg of total RNA was used to reverse transcribe to cDNA using the PrimeScript^TM^ RT reagent Kit with gDNA Eraser (Takara, Japan). RT-qPCR was conducted to assess the expression of PPARγ2, IRS1, and GLUT4 using an ABI7500 PCR system (Applied Biosystems, San Francisco, CA, United States). β-actin was used for normalization and the relative expression for each target gene was calculated using the formula 2^-ΔΔCt^. The primers used to amplify target genes and β actin were listed in Supplementary Table S1. All animal experimental protocols were carried out according to the standards of the Guide for the Care and Use of Laboratory Animals and approved by the ethics committee of Peking Union Medical College Hospital (No. XHDW-2016-001).

### Statistical Analysis

All values were expressed as mean ± standard deviation (SD) or median (interquartile range). Statistical analysis was performed by *t*-test for two groups or one-way ANOVA followed by Dunnett t (two-sided) or Dunnett t3 post-test for three or more groups. Skewed data were ln-transformed and Kruskal–Wallis test was used if data were still not normally distributed. Differences between groups were considered as statistically significant at *P* < 0.05. All statistical computations were run on SPSS 22.0 for Windows (SPSS, Inc., Chicago, IL, United States).

## Results

### Verification of pGL3-Enhancer-PPARγ2 (625 bp)-Luc Plasmid by Electrophoresis

**FIGURE 1:**
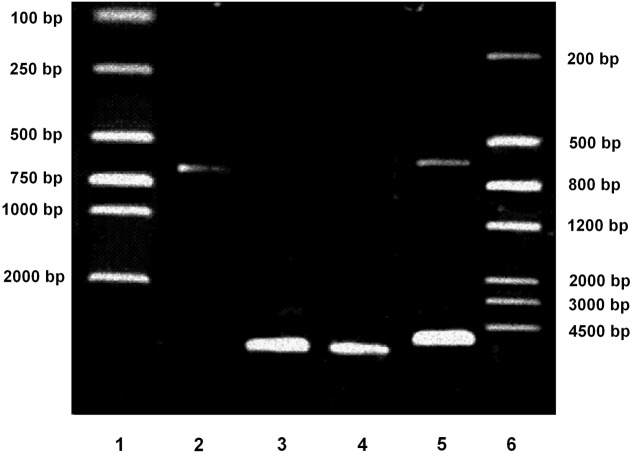
Verification of pGL3-Enhancer-PPARγ2 (625 bp)-Luc plasmid by electrophoresis. Lane 1: DNA marker DL2000. Lane 2: Inserting fragment from pGL3-PPARγ2 (625 bp)-Luc digested by KpnI and BglII (625 bp). Lane 3: Vector fragment from pGL3-Enhancer-Luc digested by KpnI and BglII (5064 bp). Lane 4: The constructed pGL3-Enhancer-PPARγ2 (625 bp)-Luc plasmid (5689 bp). Lane 5: The constructed pGL3-Enhancer-PPARγ2 (625 bp)-Luc digested by KpnI and BglII (5064/625 bp). Lane 6: DNA marker III.

The constructed pGL3-Enhancer-PPARγ2 (625 bp)-Luc plasmid were digested by KpnI and BglII. The fragments produced were electrophoresed. As shown in **Figure 1**, the digested products of the predicted lengths appeared (5064- and 625-bp). The plasmid was also verified by DNA sequencing, finally demonstrating that construction of pGL3-Enhancer-PPARγ2 (625 bp)-Luc plasmid which contained the human PPARγ2 gene 5′-promoter fragment spanning -615 to +10 bp was successful.

### Effects of Different Concentrations and Action Time of LLAE on PPARγ2 Promoter Activities in 3T3-L1 Preadipocytes

**FIGURE 2:**
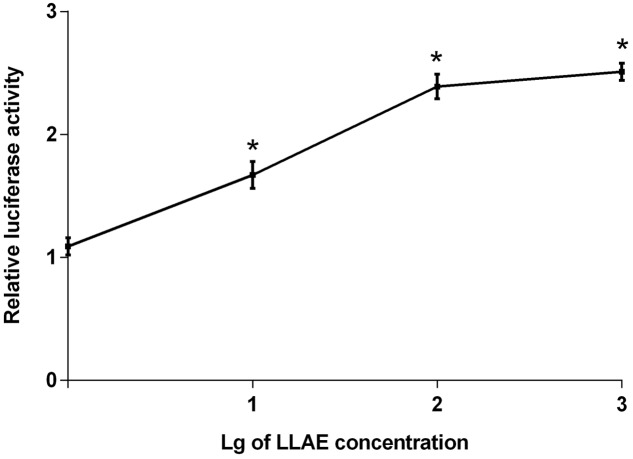
Effects of different concentrations of LLAE on PPARγ2 promoter activities in 3T3-L1 preadipocytes. Stably transfected 3T3-L1 preadipocytes were plated in 12-well plates at a density of 2.5 × 10^5^ cells/well and cultured in medium with different concentrations of LLAE (1 ∼ 1000 μg/ml) for 18 h. Then cells were lysed and the luciferase activities were measured. The relative luciferase activity was obtained in comparison with that of controls. The data represent the mean ± SD of three separate wells in three different colonies. ^∗^*P* < 0.01 vs. controls (without LLAE treatment).

The stably transfected 3T3-L1 preadipocytes were treated with 1 ∼ 1000 μg/ml LLAE for 18 h to explore the effects of LLAE on luciferase activities. As shown in **Figure 2**, 10 μg/ml LLAE significantly increased the luciferase activities in 3T3-L1 preadipocytes. The increase was further augmented with an increase of LLAE concentration. The maximal stimulatory action was noted to be 2.51 folds above controls after treating 3T3-L1 preadipocytes with 1000 μg/ml LLAE (*P* < 0.01). These findings suggest that LLAE could stimulate PPARγ2 promoter activities in a dose-dependent manner.

**FIGURE 3:**
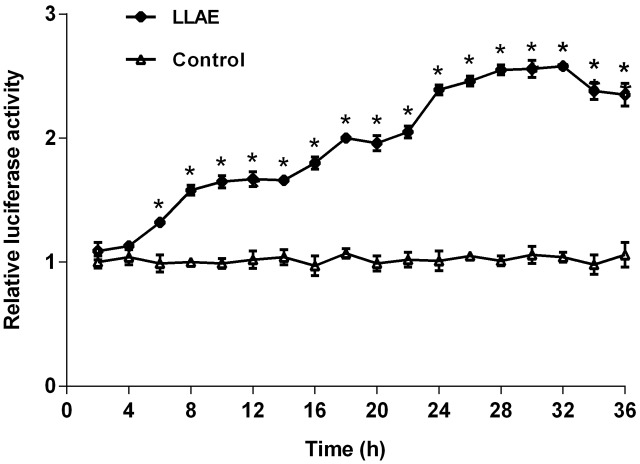
Effects of different action time of LLAE on PPARγ2 promoter activities in 3T3-L1 preadipocytes. Stably transfected 3T3-L1 preadipocytes were plated in 12-well plates at a density of 2.5 × 10^5^ cells/well and cultured in medium with 100 μg/ml LLAE for different time (2 ∼ 36 h). Then cells were lysed and the luciferase activities were measured. The relative luciferase activity was obtained in comparison with that of controls. The data represent the mean ± SD of three separate wells in three different colonies. ^∗^*P* < 0.01 vs. controls (without LLAE treatment).

In order to investigate the effects of different action time of LLAE on luciferase activities, the stably transfected 3T3-L1 preadipocytes were treated with 100 μg/ml LLAE and luciferase activities were detected every 2 h from 0 to 36 h. As presented in **Figure 3**, the relative luciferase activities in 3T3-L1 preadipocytes started to increase at 6 h and increased gradually with the prolongation of action time. The maximum effect was observed at 32 h after LLAE stimulation where it was 2.58 folds of control cells. It remained up to 36 h where it was still 2.35 folds of controls (*P* < 0.01). These data indicate that LLAE could stimulate PPARγ2 promoter activities in a time-dependent manner.

### Effects of LLAE on PPARγ2 Expression in Human Adipocytes

**FIGURE 4:**
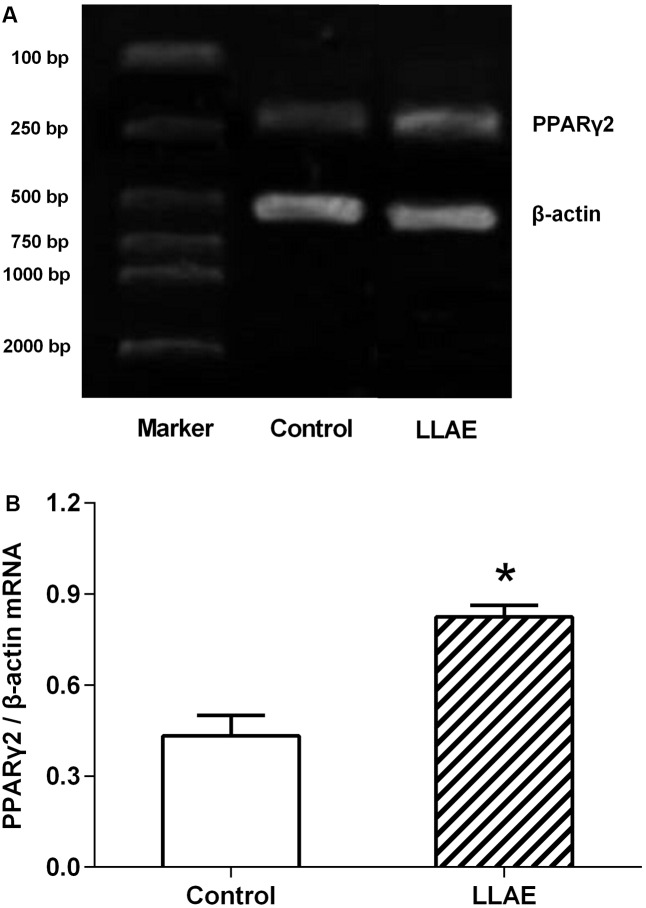
Effects of LLAE on PPARγ2 expression in human adipocytes. Human primary preadipocytes were seeded in six-well plates at a density of 5 × 10^4^ cells/well. The cells were then differentiated as described in Section “Materials and Methods.” On the sixth day of differentiation, the cells were treated with 100 μg/ml LLAE for 36 h. The total RNA was extracted and RT-PCR was performed as described in Section “Materials and Methods.” **(A)** The RT-PCR products were electrophoresed in 2% agarose gel with ethidium bromide and photographed under ultraviolet light. **(B)** PPARγ2 expression in human adipocytes treated with LLAE was calculated by the density ratio of PPARγ2 to β-actin PCR products. The data represent the mean ± SD of three separate wells in three independent experiments. ^∗^*P* < 0.01 vs. controls (without LLAE treatment).

Since LLAE could stimulate PPARγ2 promoter activities in 3T3-L1 preadipocytes as evidenced in the above experiments, whether or not it could promote the PPARγ2 expression in adipocytes? In order to answer this question, human preadipocytes preserved in our laboratory were differentiated to adipocytes according to the standard differentiation protocols as shown in Section “Materials and Methods.” On the sixth day after differentiation, cells were treated with 100 μg/ml LLAE for 36 h and then PPARγ2 mRNA levels were observed by RT-PCR. The changes of PPARγ2 mRNA levels were calculated by the density ratio of PPARγ2 to β-actin RT-PCR products in electrophoresis image. As presented in **Figure 4A**, the band density of PPARγ2 RT-PCR products was higher in LLAE treated cells than that of control cells, and it was 1.91 folds of control cells by the semi-quantitative analysis as shown in **Figure 4B** (*P* < 0.01). This result suggests that LLAE also significantly increase PPARγ2 mRNA levels in addition to its stimulatory role in PPARγ2 promoter activity.

### Effects of LLAE on Body Weight, Food Intake, and Visceral Fat Mass in HFD-Induced Obese Rats

**FIGURE 5:**
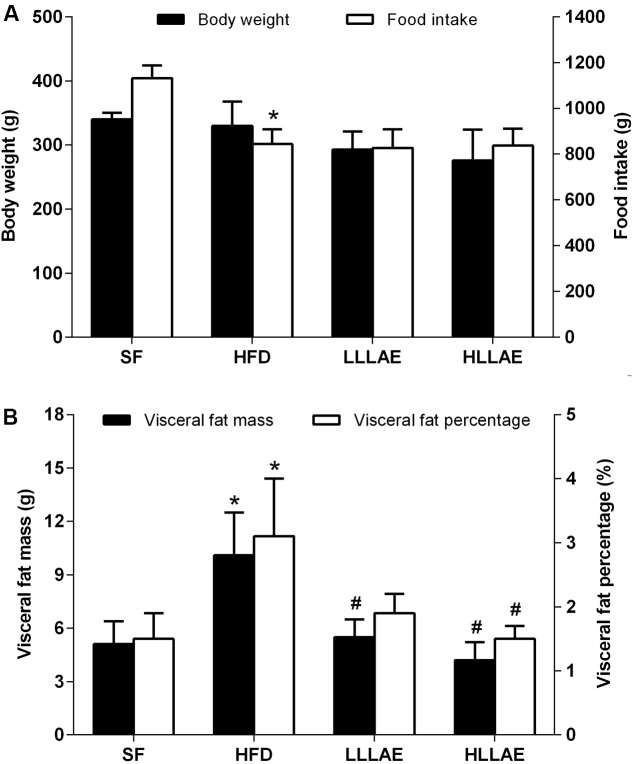
Effects of LLAE on body weight, food intake **(A)** and visceral fat mass **(B)** in HFD-induced obese rats. HFD-induced obese rats were administrated with low dose LLAE [0.5 g crude drug/(kg.d), LLLAE] or high dose LLAE [3.0 g crude drug/(kg.d), HLLAE] for 6 weeks by gavages. Body weight and food intake were recorded, and visceral fat (epididymal and perirenal fat) mass was weighted as described in Section “Materials and Methods.” The visceral fat percentage was calculated by the percentage of body weight occupied by the visceral fat mass. The data represent the mean ± SD. ^∗^*P* < 0.05 vs. SF group; ^#^*P* < 0.05 vs. HFD group.

In order to further explore the effects of LLAE *in vivo*, an animal experiment on HFD-induced obese rats was conducted subsequently. As presented in **Figure 5**, the visceral fat mass including epididymal and perirenal fat, and the visceral fat percentage (the percentage of body weight occupied by the visceral fat mass) of rats fed with HFD for six weeks were significantly increased to 1.98 and 2.07 folds of rats fed with SF (10.1 ± 2.4 g vs. 5.1 ± 1.3 g; 3.1 ± 0.9% vs. 1.5 ± 0.4%, all *P* < 0.05) although there were no notable changes of body weight observed in these two group rats. Interestingly, after administration these HFD-induced obese rats with low dose (LLLAE group) or high dose (HLLAE group) LLAE for 6 weeks, the visceral fat mass were significantly decreased by 45.5% (5.5 ± 1.0 g vs. 10.1 ± 2.4 g, *P* < 0.05) and 58.4% (4.2 ± 1.0 g vs. 10.1 ± 2.4 g, *P* < 0.05), respectively, as shown in **Figure 5B** Moreover, visceral fat percentage of rats in HLLAE group was also significantly decreased by 51.6% in comparison with that of rats in HFD group (1.5 ± 0.2% vs. 3.1 ± 0.9%, *P* < 0.05). In addition, LLAE intervention did not affect food intake of HFD-induced obese rats although there was a decrease in food intake of HFD fed rats when compared with SF fed rats as presented in **Figure 5A** (*P* < 0.05). These data show that LLAE significantly reduced visceral fat mass and visceral fat percentage in HFD-induced obese rats.

### Effects of LLAE on Biochemical Parameters, Insulin, and HOMA-IR in HFD-Induced Obese Rats

As displayed in **Table 1**, serum TC and LDL-c levels in HFD group rats were higher than that in SF group (*P* < 0.05), which were 1.95 and 4.47 folds of that in SF group, respectively. Compared with HFD group, rats in both LLLAE and HLLAE groups manifested lower fasting insulin levels (*P* < 0.05). Meanwhile, rats in HLLAE group also displayed significant decrease in HOMA-IR [3.8 (1.6, 13.8) vs. 21.1 (6.4, 35.8), *P* < 0.05] when compared with that of HFD group. However, there was no difference in FBG and serum lipid levels in LLAE intervention rats. These data indicate that LLAE significantly ameliorated insulin resistance in HFD-induced obese rats.

**Table 1:** Effects of LLAE on biochemical parameters, insulin and HOMA-IR in HFD-induced obese rats^Δ^.

	SF (*n* = 8)	HFD (*n* = 7)	LLLAE (*n* = 8)	HLLAE (*n* = 7)
FBG (mmol/L)	9.6 ± 3.5	10.7 ± 1.3	12.0 ± 0.3	11.0 ± 3.5
TC (mg/dL)	60.0 ± 17.5	116.9 ± 29.8ˆ*	112.6 ± 25.5	124.9 ± 35.8
TG (mg/dL)	44.5 ± 21.6	72.4 ± 12.5	57.8 ± 17.9	70.9 ± 37.3
LDL-c (mg/dL)	7.4 ± 2.0	33.1 ± 14.9ˆ*	30.6 ± 10.5	39.4 ± 14.6
HDL-c (mg/dL)	50.0 ± 17.0	60.9 ± 8.3	65.3 ± 8.8	60.1 ± 7.0
Insulin (pmol/L)	72.5 (26.3, 438.7)	294.5 (96.0, 416.5)	93.2 (38.1, 125.5)^#^	37.7 (27.9, 187.0)^#^
HOMA-IR	4.8 (1.9, 38.7)	21.1 (6.4, 35.8)	7.6 (3.6, 11.0)	3.8 (1.6, 13.8)^#^

**^Δ^***Values are mean ± SD or median (25th %–75th %). SF, standard food group; HFD, simple high fat diet group; LLLAE, low dose LLAE intervention group; HLLAE, high dose LLAE intervention group; FBG, fasting blood glucose; TC, total cholesterol; TG, triglycerides; LDL-c, low density lipoprotein-cholesterol; HDL-c, high density lipoprotein-cholesterol; HOMA-IR, homeostasis model assessment of insulin resistance. ^∗^*P* < 0.05 vs. SF group; ^#^*P* < 0.05 vs. HFD group*.

### Effects of LLAE on the Expression of PPARγ2, IRS1, and GLUT4 in VAT of HFD-Induced Obese Rats

**FIGURE 6:**
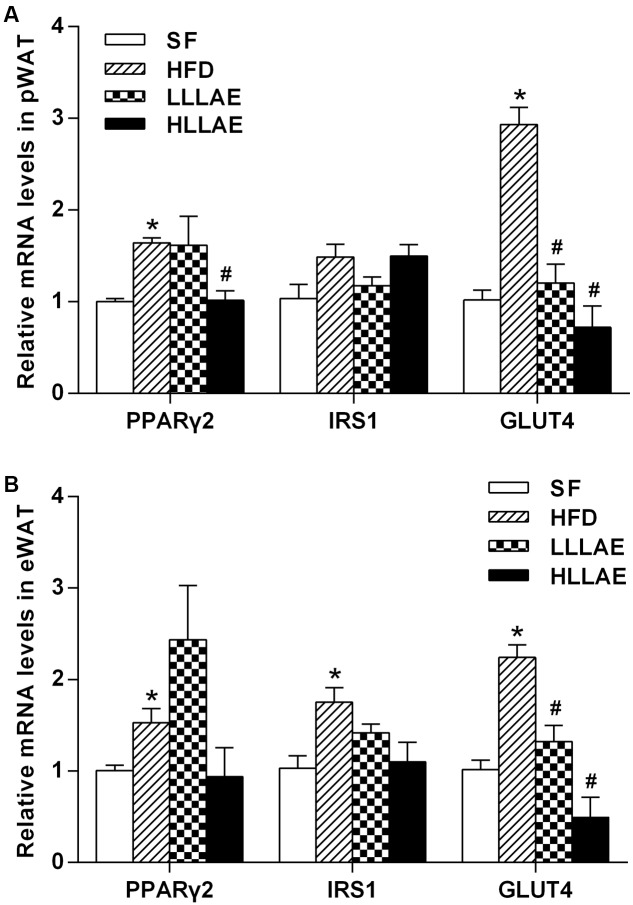
Effects of LLAE on the expression of PPARγ2, IRS1, and GLUT4 in pWAT **(A)** and eWAT **(B)** of HFD-induced obese rats. HFD-induced obese rats were administrated with low dose LLAE [0.5 g crude drug/(kg.d), LLLAE] or high dose LLAE [3.0 g crude drug/(kg.d), HLLAE] for 6 weeks by gavages. Then perirenal white adipose tissue (pWAT) and epididymal white adipose tissue (eWAT) were taken. The total RNA of adipose tissue was extracted and then RT-qPCR was performed as described in Section “Materials and Methods.” The data represent the mean ± SD. ^∗^*P* < 0.05 vs. SF group; ^#^*P* < 0.05 vs. HFD group.

As presented in **Figure 6A**, the mRNA levels of PPARγ2 and GLUT4 in pWAT of HFD-induced obese rats were notably increased to 1.64 and 2.93 folds of that in SF group (*P* < 0.05). After the administration of these obese rats with low and high dose LLAE, GLUT4 mRNA levels were significantly decreased by 59.0 and 75.4%, respectively (*P* < 0.05). PPARγ2 mRNA levels were significantly decreased by 38.4% after administration with HLLAE (*P* < 0.05). However, there was no significant difference in IRS1 mRNA levels of pWAT between these groups. As referring to eWAT, the mRNA levels of PPARγ2, IRS1, and GLUT4 in HFD group were significantly increased to 1.53, 1.75, and 2.24 folds of that in SF group (*P* < 0.05), and GLUT4 mRNA levels were also significantly decreased by 41.1 and 78.1% after LLLAE and HLLAE intervention, respectively (*P* < 0.05) as presented in **Figure 6B**. But the mRNA levels of PPARγ2 and IRS1 were not significantly changed in eWAT after LLAE intervention.

## Discussion

Lotus leaf has been reported to have some biological roles such as antioxidant, promoting lipolytic activity and up-regulating energy expenditure. However, the detailed mechanism by which lotus leaf functions is largely still unknown. Our current study for the first time demonstrated that LLAE could stimulate PPARγ2 promoter activities in 3T3-L1 preadipocytes and increase PPARγ2 gene expression in human subcutaneous adipocytes differentiated from preadipocytes. LLAE also significantly reduced visceral fat mass, decreased fasting serum insulin levels and ameliorated insulin resistance in HFD-induced obese rats, indicating the anti-obesity and anti-diabetic activity of LLAE. In accordance with our results, Ono et al. (2006) and Du et al. (2010) found that lotus leaf hot water or alcoholic extract both significantly lowered the body weight and the mass of epididymal, retroperitoneal, and parametrial adipose tissues in HFD-induced obese SD rats and mice. Studies performed by Guo et al. (2013) also demonstrated that nuciferine, a major active aporphine alkaloid from lotus leaf, significantly reduced body weight and the mass of epididymal and perirenal adipose tissues in hamsters fed with HFD. The similar anti-diabetic activity of lotus leaf was also reported by other researchers. Zeng et al. (2016) found that the treatment of rats with gestational diabetes mellitus with 50 and 100 mg/kg lotus leaf selenium-polysaccharide, a water extract of lotus leaf, for 2 weeks induced a significant decrease in FBG and insulin levels. Huang et al. (2011) reported that lotus leaf methanolic extract (100 mg/kg) also significantly decreased blood glucose levels of normal mice and HFD-induced diabetic mice. Studies conducted by Kim et al. (2013) demonstrated that oral administration of lotus leaf ethanol extract (500 mg/kg) significantly decreased the area under the glucose response curve in streptozotocin (STZ)-induced diabetic rats and 1% lotus leaf ethanol extract intervention for 7 weeks significantly reduced the plasma glucose level, HOMA-IR and HbA1C level in *db/db* mice. All these findings, together with our results, suggest that lotus leaf plays the anti-obesity and anti-diabetic roles by decreasing visceral fat and ameliorating insulin resistance.

It is well-known that PPARγ, a member of the nuclear-receptor superfamily, is considered to be the master regulator in promoting adipocyte differentiation and increasing insulin sensitization. Activation of PPARγ has been shown to promote differentiation of preadipocytes, thereby increasing the number of smaller and more insulin sensitive adipocytes (Okuno et al., 1998). Activation of PPARγ by TZD leads to increase the storage capacity of fatty acids in the adipocytes and thereby to decrease the amount of circulating fatty acids and ameliorate the insulin resistance (Siersbaek et al., 2010), which finally leads to the reduced plasma insulin and glucose levels (Olefsky, 2000; Leonardini et al., 2009; Sugii et al., 2009). Studies performed at the cellular level showed that PPARγ activation directly modulated the expression or phosphorylation of specific molecules of insulin signaling cascade to ameliorate insulin resistance (Leonardini et al., 2009). In the present study, LLAE was firstly found to significantly stimulate PPARγ2 promoter activities of 3T3-L1 preadipocytes in the dose and time-dependent manner, and LLAE also directly increased PPARγ2 mRNA levels in human subcutaneous adipocytes differentiated from the preadipocytes. These results indicate that LLAE might play an important role in ameliorating insulin resistance by stimulating PPARγ2 expression in preadipocytes and subcutaneous adipocytes.

The adipocytes from VAT are considered to be metabolically active, more insulin-resistant and sensitive to lipolysis than that from SAT (Ibrahim, 2010). The visceral fat was strongly related to many unhealthy conditions, including cardiovascular disease, insulin resistance, and type 2 diabetes (Carr et al., 2004; Finelli et al., 2013). It has been reported that PPARγ activation could greatly decrease the VAT mass (Mori et al., 1999; Miyazaki et al., 2002). After treated with troglitazone (a kind of TZD) for 6 months, type 2 diabetic patients showed a significant decrease in visceral fat area and a notable improvement in glucose metabolism (Mori et al., 1999). The similar results were also obtained by Miyazaki et al. (2002) who demonstrated that after intervention type 2 diabetic patients with pioglitazone (another kind of TZD) for 4 months, they had decreased visceral fat area and improved hepatic and peripheral insulin sensitivity. Studies performed by Gabriely et al. (2002) and Hirashita et al. (2012) showed that surgical removal of epididymal fat or together with the perinephric fat pad of obese rats led to the decrease of plasma insulin levels and the improvement of insulin sensitivity. All these results indicate that VAT in relative to SAT was bad adipose tissue and the decrease of VAT mass by internal medicine or surgical method could significantly ameliorate insulin resistance. In our present study, visceral fat mass, fasting serum insulin level and HOMA-IR of HFD-induced obese rats were also significantly reduced after LLAE intervention for 6 weeks, suggesting that LLAE could ameliorate insulin resistance by reducing VAT mass of HFD-induced obese rats.

Glucose transporter 4 is one of the sugar transporter proteins that catalyze hexose transport across cell membranes (Huang and Czech, 2007). It is predominantly expressed in muscle cells and adipocytes. The GLUT4 glucose transporter is a major mediator of glucose removal from the circulation to adipocytes. About 50% of the glucose taken up by the adipocytes is normally used to synthesize the glycerol and fatty acid components of triacylglycerol (Festuccia et al., 2009). Furthermore, PPARγ is capable of activating GLUT4 gene expression during adipogenesis (Wu et al., 1998). It was reported that higher PPARγ2 mRNA levels of VAT were accompanied by higher GLUT4 mRNA levels as well as higher capacity to store triacylglycerol and larger adipocyte size in Wistar rats (Palou et al., 2009). These results indicated that the activation of PPARγ2 in VAT could increase GLUT4 expression to transport more glucose into visceral adipocytes thus increasing their fat accumulation. In the current study, HFD-induced obese rats showed increased mRNA levels of PPARγ2 and GLUT4 in VAT as well as an increase of visceral fat mass compared with rats fed with SF, indicating that there was more glucose uptake and lipogenesis in visceral adipocytes in obese rats. In accordance with our results, MacLaren et al. (2007) found that GLUT4 also had a higher expression in omental adipose tissue from insulin-resistant obese subjects when compared with insulin-sensitive subjects, suggesting the increased expression levels of GLUT4 might also indicate a compensatory mechanism under the situation of insulin resistance. Interestingly, the increased expression of PPARγ2 and GLUT4 in VAT of these obese rats was significantly suppressed by LLAE intervention in our present study. These results demonstrate that LLAE could suppress PPARγ2 expression, then decrease GLUT4 expression and inhibit glucose uptake, further reduce substrate supply for triacylglycerol synthesis in VAT adipocytes and finally decrease the visceral fat mass in obese rats. Similarly, studies performed by Lee et al. (2015) also founded that kaempferol, a compound isolated from the methanolic extract of Nelumbo nucifera stamens, significantly suppressed GLUT4 expression and inhibited glucose uptake, further decreased triacylglycerol accumulation in adipocytes. Besides, nuciferine and pronuciferine, the main active components of lotus leaf, were also reported to notably decrease lipid droplets and intracellular triglyceride contents by activating the AMPK signaling pathway in mature 3T3-L1 adipocytes (Ma et al., 2015).

Additionally, we found serum TC and LDL-c levels of rats fed with HFD were higher than those fed with SF. However, the reduction of TC and LDL-c levels was not observed after LLAE administration. On the contrary, studies performed by Du et al. (2010) and Guo et al. (2013) found that lotus leaf hot water extract or its major active aporphine alkaloid nuciferine significantly reduced the serum TG, TC and LDL-c levels in HFD fed SD rats or hamsters. 1% lotus leaf ethanol extract or 50 or 100 mg/kg lotus leaf selenium-polysaccharide also significantly elevated HDL-c levels in *db/db* mice and gestational diabetes mellitus rats in addition to its role in reducing TG, TC, and LDL-c levels (Kim et al., 2013; Zeng et al., 2016). The different results between ours and literatures may be explained by the different concentrations and action time of lotus leaf or different origins and extraction methods of lotus leaf. Different animal models used in the experiment may also be one reason for these differences.

This is the preliminary study to firstly demonstrate that LLAE could directly stimulate PPARγ2 promoter activities in preadipocytes and increase PPARγ2 gene expression in subcutaneous adipocytes. Meanwhile, LLAE significantly ameliorated insulin resistance of HFD-induced obese rats, and its beneficial role was obtained possibly by reducing visceral fat mass through suppressing the expression of PPARγ2 and GLUT4 in VAT. Further studies need to be done for elucidating the effects of LLAE on the proliferation and differentiation of preadipocytes, especially preadipocytes from VAT, and the effects of LLAE on SAT mass of HFD-induced obese rats. Overall, this is a promising beginning which demonstrates that PPARγ2 is an important factor involved in the mechanism by which LLAE reduces visceral fat mass and ameliorates insulin resistance in obese rats.

## Author Contributions

KY did the cell experiments, analyzed data and wrote the primary manuscript. HZ designed the experiments and revised the primary manuscript. JX did the cell experiments and animal experiments. HP supervised the experiments. NL, LW, and HY helped to treat the animals and supervised the biochemical parameters measurements. ML analyzed data. FG designed the experiment, supervised the whole experiments and revised the primary manuscript.

## Conflict of Interest Statement

The authors declare that the research was conducted in the absence of any commercial or financial relationships that could be construed as a potential conflict of interest. The reviewer MH and handling Editor declared their shared affiliation, and the handling Editor states that the process nevertheless met the standards of a fair and objective review.

## Abbreviations

FBS: fetal bovine serum
GLUT4: glucose transporter 4
HFD: high fat diet
HLLAE: high dose LLAE
HOMA-IR: homeostasis model assessment of insulin resistance
IRS1: insulin receptor substrate 1
LLAE: lotus leaf aqueous extract
LLLAE: low dose LLAE
PPARs: peroxisome proliferative activated receptors
SF: standard food
TCMs: traditional Chinese medicines
TZD: thiazolidinedione
VAT: visceral adipose tissue.
