# Supplementary material for: Lotus Leaf Aqueous Extract Reduces Visceral Fat Mass and Ameliorates Insulin Resistance in HFD-Induced Obese Rats by Regulating PPARγ2 Expression
Source: Front Pharmacol. 2017 Jun 23;8:409. doi: 10.3389/fphar.2017.00409 (PMC5481353; doi:10.3389/fphar.2017.00409)
Supplement: Supplementary file 1 [file Table_1.DOCX]

# Supplementary Material

**Table S1: Primers for RT-PCR and RT-qPCR**

| Human PPARγ2 | F: 5’-TTATGGGTGAAACTCTGGG-3’ |
| --- | --- |
|  | R: 5’-GAAATGCTGGAGAAGTCAAC-3’ |
| Human β actin | F: 5’-GTGGGGCGCCCCAGGCACCA-3’ |
|  | R: 5’-CTTCCTTAATGTCACGCACGATTTC-3 |
| Rat PPARγ2 | F: 5’-GCTGTGAACCACTAATATCCAAG-3’ |
|  | R: 5’-ATGGCATCTCTGTGTCAACC-3’ |
| Rat IRS1 | F: 5’-TGAGAGCGGTGGTGGTAAG-3’ |
|  | R: 5’-ATGAGTAGTAGGAGAGGACAGG-3’ |
| Rat GLUT4 | F: 5’-CCAGTATGTTGCGGATGC-3’ |
|  | R: 5’-GGAAGGTGAAGATGAAGAAGC-3’ |
| Rat β actin | F: 5’-ATCGGCAATGAGCGGTTC-3’ |
|  | R: 5’-ACTGTGTTGGCATAGAGGTC-3’ |

F: forward primer, R: reverse primer
